# Symptomatic Bilateral Aberrant Course of the Internal Carotid Arteries Presented as a Non-pulsatile Mass in the Posterior Pharynx: A Case Report and Literature Review

**DOI:** 10.7759/cureus.65717

**Published:** 2024-07-30

**Authors:** Fatimah Alzubaidi, Mohammed H Aldebasi, Hamza A Alandijani, Abdulrahman F Kabli, Mohammad S Jalaladdin, Lina F Serhan, Adari Alqurashi

**Affiliations:** 1 Head, Neck and Skull Base Center, King Abdullah Medical City, Makkah, SAU; 2 Department of Ophthalmology, Ministry of National Guard - Health Affairs, King Abdullah International Medical Research Center, Riyadh, SAU; 3 Otolaryngology, National Guard Hospital, Medina, Medina, SAU; 4 Otolaryngology - Head and Neck Surgery, Makkah Health Cluster, Makkah, SAU; 5 Department of Medicine and Surgery, College of Medicine, Umm Al-Qura University, Makkah, SAU; 6 Faculty of Medicine, Almaarefa University, Riyadh, SAU; 7 Otolaryngology - Head and Neck Surgery, Maternity and Children Hospital, Makkah, SAU

**Keywords:** rare, symptomatic, non-pulsatile mass, posterior pharynx, internal carotid arteries

## Abstract

The atypical congenital pathway of the internal carotid artery (ICA) is an uncommon anatomical variation with a very low prevalence. The medialization of the internal carotid artery is regarded as an infrequent manifestation. The internal carotid artery may be displaced at the level of the pharyngeal wall, leading to the enlargement of connective tissue in the lateral pharynx and retropharyngeal areas. A 76-year-old male patient with a history of weakness on the left side of his body, difficulty swallowing, and speech difficulties was sent to the otorhinolaryngology department because of pain in his throat. He underwent several unsuccessful attempts at the insertion of a nasogastric tube (NGT), which was eventually done with considerable difficulty.

Upon evaluation, the individual displayed regular speech and a strength rating of 4/5 in both of his left limbs. Upon examination of the throat, a significant non-pulsating edema was observed in the right retropharyngeal area, pushing the right tonsil anteromedially. A posterior pharyngeal mass was observed during fiberoptic laryngoscopy. The Doppler examination of the carotid arteries yielded definitive results. Computed tomography angiography (CTA) showed the common carotid arteries via a retropharyngeal route. It is clinically significant to identify variations in the course of the internal carotid artery, particularly those located near the submucosal area of the pharynx. This is because there is a higher risk of injury during procedures involving manipulation of the pharynx, such as intubation, insertion of a nasogastric tube, or surgeries in the internal carotid artery region.

## Introduction

The congenital aberrant course of the internal carotid artery (ICA) is an infrequent anatomical variant. Several types of anomalies have been described in the literature. With an incidence of 0.2%, medialization of the ICA is considered rare and has a rare presentation [1,2]. Displacement of the ICA may occur at the level of the pharyngeal wall, causing the expansion of connective tissue in the lateral pharynx and retropharyngeal areas. Some patients may present with symptoms such as dysphagia, dystonia, fullness of throat, or pulsatile swelling. This variant may be recognized incidentally during the clinical assessment of otorhinolaryngological cases [1]. Undiagnosed cases can result in fatal complications and uncontrolled bleeding.

## Case presentation

A 76-year-old man with a known case of controlled diabetes and hypertension presented to our hospital with a history of left-sided weakness, dysphagia, and slurred speech. The patient was referred to otorhinolaryngology (ORL) because of throat pain. He underwent multiple attempts of failed nasogastric tube (NGT) insertion, which was eventually successful with difficulty. On examination, the patient was vitally stable on an oxygen cannula because the operative bouts of unexplained hypoxemia decreased to 89% improvement on oxygen supplements, and he had normal speech with a power of 4/5 in both left limbs.

**Figure 1:**
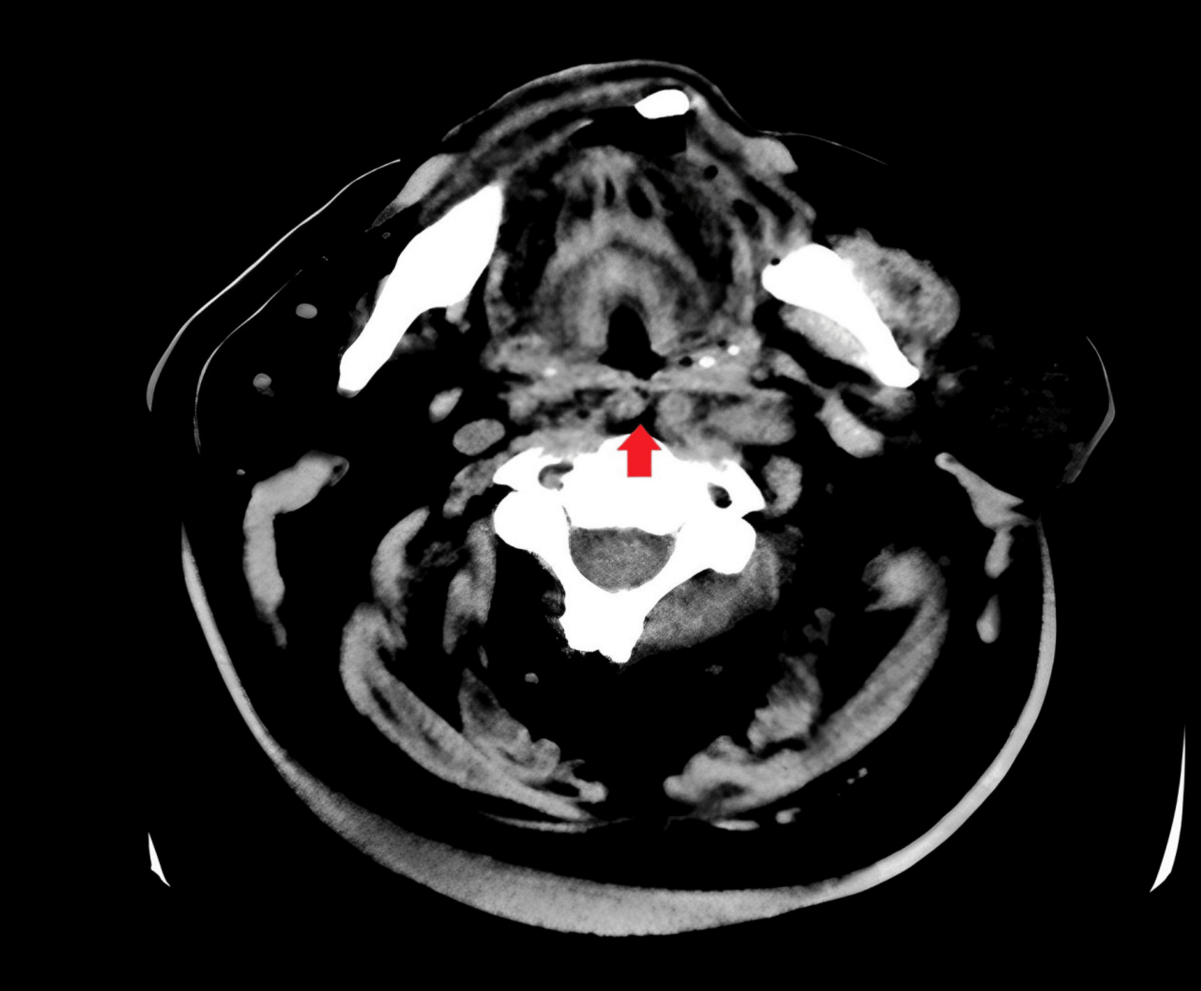
Computed tomography angiogram (CTA) showing bilateral aberrant course of the internal carotid arteries

However, a throat examination revealed a large right retropharyngeal non-pulsatile swelling pushing the right tonsil anteromedially. Fiber optic laryngoscopy showed bilateral mobile vocal cords with no abnormalities noted apart from the posterior pharyngeal mass, and normal swallowing was observed. Barium swallowing documented normal swallowing with no aspiration or laryngeal penetration. The Doppler carotid arteries were clearly conclusive. Computed tomography angiography (CTA) revealed mild atherosclerotic changes in the aortic arch; however, the major branches appeared well opacified and patent, with no significant stenosis. The common carotid arteries showed a retropharyngeal course that started from the level of the clavicles, with both arteries traveling in the retropharyngeal region with proximity to each other, approximately 7 mm apart, with both arteries separating and moving laterally at the level of C1 to continue as the internal carotid arteries (Figure 1). 

Furthermore, the intracranial parts of the internal carotid arteries showed moderate atherosclerotic changes with a mildly calcified atheroma, especially on the right side. However, the distal artery showed mild opacification, and the circle of Willis appeared unremarkable. We documented the findings as rare, necessitating carefulness in any procedure near the pharyngeal area. The patient received only 1 gram of paracetamol through IV every eight hours for throat pain. The patient was discharged by the ORL team, and precautions were given to the patient regarding any intervention in or examination of the pharyngeal area. No follow-up was needed with us. 

## Discussion

The ICA emanates from the bifurcation of the common carotid artery at the level of C3-C4 in adults and C2-C3 in children. The ICA is divided into seven segments: cervical, petrous, lacerum, cavernous, clinoid, ophthalmic, and communicating, which largely supply the intracranial and extracranial structures [3]. The cervical part of the ICA ascends within the carotid sheath until it reaches the carotid canal in the petrous bone of the skull base [4]. In general, the extracranial part of the ICA course is straight without branching. The congenital variation of the ICA is classified into “tortuosity,” which is defined as any alteration of the artery course, e.g., elongation, or “kinking,” which is an acute angulation in the course of the artery [5]. Several studies have shown the medialization of the internal carotid artery, which may course submucosally in the pharyngeal wall [6]. Galletti et al. reported five cases of asymptomatic bulging pharyngeal masses [1,7]. A study showed that pharyngeal wall pulsation was detected in 1-16% of the population, mostly on the right side, with a female predominance [2].

The underlying cause of such cases may worsen owing to the aging process, as in hypertension-related changes and atherosclerosis. Patients’ presentations may vary; some present with dysphagia, abnormal sensation in the throat, hoarseness of the voice, or are asymptomatic [1,8]. Recognition of such a displacement in the ICA, which lies in close proximity to the submucosal area, is of clinical importance because of the increased risk of injury through intubation or surgery around the ICA region [1]. Furthermore, a puncture may also follow drainage of an abscess in the retropharyngeal area, and more importantly, in less invasive procedures such as adenoidectomy, tonsillectomy, or even during scoping in general, an otorhinolaryngology examination may lead to catastrophic uncontrolled bleeding [9,10]. In a retrospective study, the data of patients who died after tonsillectomy were reviewed, and strategies were developed to prevent complications, such as close inspection of the nasopharynx immediately before adenoidectomy and curettage [9]. Another study suggested a classification system for ectopic ICA based on the mucosa of the pharynx [11]. Different case reports and series that have reported on the different locations of the ICA and the presentations of the patients are summarized in Table 1. 

**Table 1:** Summary of different reported cases of the aberrant course of the internal carotid arteries

Study	Sex	Age	Location	Sign and symptoms	Follow-up
Galletti et al., 2002 [1]	Male	78, 63, 57, 32, and 8 years	Back to right tonsillar pillar and in one case left	Asymptomatic except for one complaint of dysphagia	Not mentioned
Ziółkowska et al., 2017 [5]	Female	63 years	Posterior wall of epipharynx	Sinus headache, fever, dysphonia, and episodes of dyspnoea	Not mentioned
Gupta et al., 2013 [6]	Female	28 years	Oropharynx	Cervical lymphadenopathy and sore throat	Not mentioned
Prokopakis et al., 2008 [8]	Male	58 years	Oropharynx	Dysphagia and malaise	Not mentioned

## Conclusions

Recognition of ICA course variations, especially proximal to the submucosal area of the pharynx, is clinically significant because of the increased risk of injury through pharyngeal manipulation, such as intubation, NGT insertion, or surgery around the ICA region. Different variations of ICA that are reported should be considered, along with different patient presentations, during any procedure near the pharynx and oropharynx. We aim to report this case to increase physician awareness regarding the different anatomical positions of ICA.
